# Caring for Pregnant Patients with Cancer: A Framework for Ethical and Patient-Centred Care

**DOI:** 10.3390/cancers16020455

**Published:** 2024-01-21

**Authors:** Alma Linkeviciute, Rita Canario, Fedro Alessandro Peccatori, Kris Dierickx

**Affiliations:** 1 Fertility and Procreation Unit, Division of Gynecologic Oncology, European Institute of Oncology, IRCCS, 20141 Milan, Italy; 2 Cancer Metastasis i3S-Institute for Research & Innovation in Health, R. Alfredo Allen 208, 4200-135 Porto, Portugal; rita.canario@ipatimup.pt; 3 Research Centre, Portuguese Oncology Institute of Porto, 4200-072 Porto, Portugal; 4 ICBAS—School of Medicine and Biomedical Sciences, University of Porto, R. Jorge de Viterbo Ferreira 228, 4050-313 Porto, Portugal; 5 Centre for Biomedical Ethics and Law, KU Leuven, 3000 Leuven, Belgium; kris.dierickx@kuleuven.be

**Keywords:** cancer during pregnancy, ethics, guidelines, patient centricity, patient engagement

## Abstract

**Simple Summary:**

Cancer treatment during pregnancy can raise many difficult questions. Currently available clinical practice guidelines offer very limited ethical guidance for healthcare professionals. This article offers a theoretical framework and a practical ethics checklist for ethical and patient-centred care. It takes a holistic view to patient treatment, care and counselling that emphasises the need to recognise the relational context of individual patient’s autonomy; balance maternal and foetal beneficence obligations; balance maternalistic and relational approaches to evidence-based personalised patient care; consider protection of the vulnerable in light of responsibilities towards the unborn; and ensure reasonable and just resource allocation. At the moment, very few studies have explored clinicians’ attitudes and patients’ experiences when cancer treatment is delivered during pregnancy. Therefore, future work will require patient engagement to develop ethical guidance in this setting.

**Abstract:**

(1) Background: Caring for pregnant cancer patients is clinically and ethically complex. There is no structured ethical guidance for healthcare professionals caring for these patients. (2) Objective: This concept paper proposes a theoretically grounded framework to support ethical and patient-centred care of pregnant cancer patients. (3) Methodological approach: The framework development was based on ethical models applicable to cancer care during pregnancy—namely principle-based approaches (biomedical ethics principles developed by Beauchamp and Childress and the European principles in bioethics and biolaw) and relational, patient-focused approaches (relational ethics, ethics of care and medical maternalism)—and informed by a systematic review of clinical practice guidelines. (4) Results: Five foundational discussion themes, summarising the key ethical considerations that should be taken into account by healthcare professionals while discussing treatment and care options with these patients, were identified. This was further developed into a comprehensive ethics checklist that can be used during clinical appointments and highlights the need for a holistic view to patient treatment, care and counselling while providing ethical, patient-centric care. (5) Conclusion: The proposed framework was further operationalised into an ethics checklist for healthcare professionals that aims to help them anticipate and address ethical concerns that may arise when attending to pregnant cancer patients. Further studies exploring clinicians’ attitudes towards cancer treatment in the course of pregnancy and patient experiences when diagnosed with cancer while pregnant and wider stakeholder engagement are needed to inform the development of further ethical, patient-centred guidance.

## 1. Introduction

Providing care to pregnant cancer patients is complex as they present healthcare needs and decisional dilemmas that encompass distinct and often overlapping dimensions [1]. Looking after these patients raises not only clinical challenges, such as choosing the most suitable treatment regiment, time of delivery and breastfeeding support following the birth of an infant, but also many concerns and decisional challenges for the patients [2] and ethical challenges for the clinical teams [3].

Treatment choices are challenging as the evidence derived from prospective clinical trials is scarce due to study design and participant recruitment difficulties in this setting. Even though it is known that pregnancy does not impair the clinical outcomes of patients who have undergone curative treatment for some cancers [4,5], less data are available regarding its impact on those with advanced cancers which require multimodal treatment protocols [1,6]. Moreover, some cancers might be more difficult to manage during pregnancy and some treatments may be not feasible during pregnancy. For example, uterine cancer poses the challenge that the organ affected by cancer is also the one bearing the pregnancy [7,8] and chemotherapy given during the first trimester may be associated with a higher rate of foetal malformations and pregnancy complications [9]. Therefore, treatment decisions always need to be informed by the gestational age of the foetus as well as the site, stage and biological features of the tumour (Table 1). Recent recommendations suggest that radiotherapy is technically feasible during pregnancy, specifically for tumours that are remote to the foetus, such as breast and head and neck cancer [10]. Immunotherapies and targeted agents are usually contraindicated in pregnancy with some reports of congenital hypothyroidism [11] or severe immune-mediated enteritis [12] following in utero exposure to anti-PD1. On the contrary, other reports present positive maternal outcomes after immunotherapy exposure [13,14,15]. Further reviews also suggest that targeted therapies (e.g., trastuzumab) in the first trimester are less likely to lead to complications [16], and their use during pregnancy might be possible under close monitoring [17], but the risk of pregnancy and foetal complications remains high [18]. Thus, standard treatments cannot be always given to pregnant cancer patients, reinforcing the difficulty of managing such patients.

**Figure 1:**
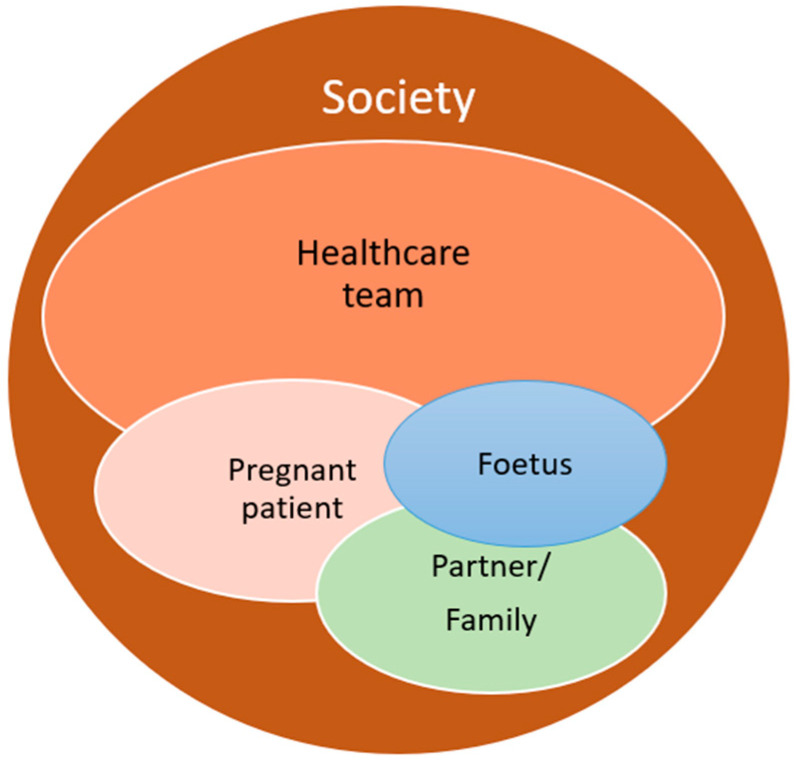
Conflicting interests in cancer during pregnancy care. The bubble sizes and colours in the above figure are not intended to rank the importance of the affected stakeholders.

The decision-making process in this setting is also challenging for patients and often raises ethical dilemmas. Patients might need support with attending hospital appointments and enduring treatment procedures, while at the same time preparing for the arrival of the new family member and taking care of an infant. An extra layer of complexity stems from the idiosyncratic nature of this condition, as the patient herself is not the only player involved or affected (Figure 1). Ethical questions raised by the healthcare team, patients and/or their families are not always the same and might be conflicting, such as when one party does not feel comfortable with care decisions that are either taken or desired by another party involved [33,34]. For these reasons, taking care of pregnant cancer patients requires a multidisciplinary clinical team that should also include decisional counsellors [35,36], psychologists and ethicists [37], in addition to the oncological core medical team [1,38,39,40].

To provide patient-centric, ethically and legally informed care for pregnant cancer patients, a holistic view should be taken towards patient’s treatment, care and counselling. It is essential to consider individual circumstances of each pregnant cancer patient where each patient is seen as a person embedded in the realities of their lives and the changes that a cancer diagnosis brings to themselves and their pregnancy care. Currently, only limited ethical guidance is available for clinicians with very few resources presented in a structured and consistent manner [39,41], lacking guidelines dedicated to identifying, addressing and managing ethical issues and concerns in cancer during pregnancy care. Available resources integrate information from the guidelines focused on clinical aspects of treating different cancer types during pregnancy and are supplemented by some clinical and bioethics experts’ input. This guidance is also mostly based on references to the biomedical ethics principles, which is a significant limitation of ethical guidance available in this field.

There is an urgent need for structured ethical guidance tools for healthcare professionals to help them address existing and potential ethical issues that arise while taking care of pregnant cancer patients. Therefore, this paper aims to lay a foundation for such guidance by proposing a theoretically informed framework focused on ethical, patient-centred care of pregnant cancer patients and offering an ethics checklist for healthcare professionals to support the decision-making process in treatment and the care of pregnant cancer patients. The ethics checklist is constructed with healthcare professionals as intended end users in mind and is expected to serve as a tool to support shared decision making in cancer treatment, care and patient counselling during pregnancy. A research team that includes two clinicians involved in the treatment of these patients and two experts in bioethics used inductive and deductive reasoning while analysing ethical models available to guide cancer treatment and care during pregnancy, which was informed by a systematic review that compiled ethical guidance in published guidelines regarding cancer treatment during pregnancy [39].

## 2. Ethical Models Applicable to Cancer Care during Pregnancy

There are many ethical guidance models that can be applied to cancer care during pregnancy. However, none of them appear to adequately address all ethical issues arising in these circumstances. Four biomedical ethics principles developed by Beauchamp and Childress [42] and the European principles in bioethics and biolaw [43,44] were used as a starting point (Table 2).

Since caring for pregnant cancer patients also requires consideration of the patient’s relationships—including the patient’s perceived relationship with the foetus, partner, other children (if present), parents, relatives, friends and wider community, as well as ethno-socio-cultural and political environment [34]—elements of ethics of care [45,46], relational ethics [47,48] and medical maternalism [49,50] were also considered. These relational, patient-focused approaches were used as adjuncts to specify the principles-based guidance and to illustrate how the proposed theoretical framework and subsequent ethics checklist can work in already existing healthcare structures by supplementing rather than challenging already established patient care processes and services.

**Table 2:** Foundational models for ethical, patient-centric care of pregnant cancer patients.

	Ethical Models Used to Develop the Guidance	Model Description and Specification	Key References
**Principle-based approaches**	Four principles for biomedical ethics (Georgetown principles) by Beauchamp and Childress	Respect to patient’s autonomy, including relational aspects Nonmaleficence: avoiding harm before doing good Beneficence: maximising the benefit for the pregnant patient and developing foetus Justice: considering a big picture and a broader context	[42]
	European principles of bioethics and biolaw presented by Rendtorff	Autonomy: individual freedom to make choices Dignity: moral responsibility to human life Integrity: right to bodily integrity, right to refuse treatment Vulnerability (respect to vulnerability): recognising human vulnerabilities, protecting vulnerable groups	[43,44]
**Relational, patient-focused approaches**	Relational ethics	Trusted relationship building with the patient Patient-centric approach to patient care Interdependency and freedom Emotions and reason	[47,48]
	Care ethics (ethics of care)	Compassion to patient’s suffering Presence in patient’s unique situation, active listening Empathy to patient’s feelings and circumstances Recognition of a patient as fellow human being	[45,46]
	Medical maternalism	Shared decision making Accessible evidence-based information Conversation and understanding of patient’s circumstances and best interest Patient guidance through clinical advice and reason	[49,50]

### 2.1. Principle-Based Approaches

Classical biomedical ethics principles (respect for patient’s autonomy, nonmaleficence, beneficence and justice) developed by Beauchamp and Childress [51], also known as the Georgetown principles, are both directly and indirectly referenced by clinical practice guidelines for cancer treatment during pregnancy [39]. The European approach to biomedical ethics offers autonomy, dignity, integrity and vulnerably as core guiding principles, which are to be considered in the broader context where a patient is seen as being part of a wider human ecosystem [44]. Therefore, respect for patients’ autonomy, which requires due attention to be given to the individual patient’s views and wishes (as well as their participation in decision making) is not limited to the patient alone. Beauchamp and Childress recognise that following the respect for autonomy principle without considering the context is problematic because patients do not live in isolation and out of context. Their treatment choices might affect other people in their lives, and the patient’s choices can also be influenced or even directed by people that pregnant cancer patient considers significant [42].

Therefore, in addition to autonomy, it is suggested that in the European context, dignity should further support the moral responsibility to human life [44], which is perceived broadly. It is not intended to escalate disagreements on how and whether the life of the unborn should be protected. Dignity, together with bodily integrity, overarch classical nonmaleficence and beneficence principles proposed by Beauchamp and Childress. Moreover, vulnerability (or respect for vulnerability) has been emerging as a wider recognised principle in biomedical ethics [52,53], which could be distinguished either as a requirement to protect vulnerable groups [54] or as merging with other principles, such as the principles of nonmaleficence, autonomy, dignity and bodily integrity [55]. Some authors also mention “vulnerability to co-creation”, especially in contexts surrounding the reproductive decisions, parental roles and dependent status of women [56], where their personal identity is mirrored through their relationships with others [57,58].

Principles on their own, however, can be too rational and too rigid for addressing ethical issues in everyday clinical practice [59] as—for example—is the doctrine/rule of double effect referenced by Beauchamp and Childress in regards to maternal–foetal conflict [42]. Maternal–foetal conflict is sometimes presented as deliberate harm to the developing foetus caused by its mother’s ignorance or unwillingness to adhere to standard pregnancy care [60], which is not necessarily the case when cancer is diagnosed during pregnancy. Principles can also fall in a conflict with each other, especially respect for patients’ autonomy and clinicians’ beneficence obligations [51]. Moreover, the principled approach has been shown to be less culturally neutral than it might initially appear, especially in a non-Judeo–Christian context [61]. Therefore, principle-based models need to be supplemented with other ethical models in order to address the ethical and patient-centred care needs. Some attempts have been made to blend principle-based approaches with casuistry when resolving clinical cases [62], which has been shown to be a valuable addition to technological decisional support tools aimed at patient-centred healthcare [63].

### 2.2. Relational, Patient-Focused Approaches

Shared decision making and care of pregnant cancer patients can be a very intense, emotional and psychologically demanding task for the healthcare team. It might challenge clinical teams’ attitudes towards the patient, their circumstances and even relationship with the patient. Indeed, the pregnant cancer patient’s relationship with the clinical team is of pivotal importance, requiring recognition of the patient’s unique situation; understanding of their individual circumstances; and empathy with their clinical, moral and practical concerns. Relational ethics can support clinicians with a patient-centred approach to patients by guiding the relationships toward empathy, attempting to understand patients’ emotions and reasoning [47].

The ethics of care further specify that compassion, presence, empathy and recognition of a patient as a fellow human being play a significant role in building trusted relationships between healthcare professionals and their patients [64], which is regarded with high importance in midwifery [46].

Medical maternalism is one more patient-focused approach emerging in contemporary bioethics [50]. It considers patients’ autonomy in a relational context and encourages patient support by the clinical team, where patients are provided correct, up-to-date and easy to understand information in order to guide them through the decision-making process with their best interests in mind.

Caring for pregnant cancer patients can have further relational and care obligation complexities. Some healthcare professionals might feel an obligation to protect the developing foetus and might consider it a separate patient [65,66]. Such protection, however, can only be achieved if pregnant patients perceive foetal interests the same way as the clinical team does and are willing to collaborate with the clinicians. Some jurisdictions might have legal frameworks governing pregnancy care and restricting pregnancy termination. A recent example from the United States in the *Dobbs* vs. *Jackson* case shows that cancer treatment options might be restricted for pregnant patients [38,67], despite the historic trend denying foetuses a legal entity status until they are born [68].

Relational, patient-focused approaches to patient care can fall short of being universally applicable as they mostly describe the practice of providing care [69]. Such approaches are based on forming trusted relationships with the patient, but they do not offer a clear set of rules to follow which could be applied in different patient care scenarios. Furthermore, some suggest that care ethics and relational approaches to patient care are solely based on Western perceptions of patient care and lack cultural representativeness and inclusiveness [70].

## 3. Discussion Themes for Ethical, Patient-Centred Cancer Care during Pregnancy

Clinical conversations concerning the ethics of cancer treatment and care during pregnancy remains theoretical, focusing on physical care and technical interventions giving just a brief, non-structured concern for ethical issues, which could arise in clinical practice. Emerging data on cancer treatment and pregnancy compatibility seem to present this phenomenon as any other type of illness, which technically could be successfully managed from a clinical point of view. Hence, the patient-centric aspects concerning emotional, psychological, ethical, spiritual, social, cultural and relational concerns experienced by those affected by a cancer diagnosis in the course of pregnancy remain on the margins [40]. While considered more in depth at times of clinical uncertainty, ethical and psychosocial concerns tend to be pushed to the background in the presence of more reassuring clinical evidence [39]. Therefore, based on the selected ethical models presented in Table 2, five key themes were identified as essential for everyday oncology practice when cancer treatment and care is provided during pregnancy (Table 3).

### 3.1. Recognising Relational Context of Individual Patient’s Autonomy

Patient autonomy in the context of cancer during pregnancy needs to be framed within the individual circumstances in which the pregnant patient expresses their relational autonomy but needs also to be supported through a caring approach to building trusted relationships between the patient and the healthcare team. The maternalistic approach to patient care can also support empowering the patient to make informed decisions in a relational context. It would require taking into consideration the patient’s circumstances as a whole and not distinguishing strictly between the clinical and social parts of patient care by asking “Now that we have this diagnosis and given your circumstances, what do you think would be best for you?” rather than “What is your treatment choice?”, thus crystallising their own preferences, expectations and perceptions on what would count as a desirable outcome in their individual situation [37,71,72,73].

Pregnant cancer patients are neither just cancer patients nor just pregnant patients. They might have wide social ties and be embedded in their surroundings. They can identify themselves through various social roles (e.g., partner, mother, friend, community member) and choose to exercise their autonomy in light of their life experiences, expectations and relationships [57,58]. A good number of pregnant cancer patients will want the best outcome for the foetus as much as a good health outcome for themselves, so they can be there for a child that the foetus is going to become and for other people significant to their lives.

Nevertheless, it should be recognised, acknowledged and respected that some patients genuinely do not want to be active actors in medical decision-making processes and prefer to be guided by the clinical team attending to their care, which would require a purely maternalistic approach to the care of these patients.

### 3.2. Balancing Maternal and Foetal Beneficence

This approach can be supported by propositions on patient care found in care ethics and medical maternalism. It requires physicians to be informed about the latest evidence-based information and ongoing research on cancer treatment during pregnancy, maternal and foetal outcomes, and remaining uncertainties, especially concerning the long-term paediatric and maternal outcomes in particular circumstances. Some clinical practice guidelines clearly state that maternal beneficence should prevail in some circumstances, while many others suggest that an equilibrium of maternal benefit and foetal protection should be sought when administering cancer treatment to a pregnant patient [7,74,75,76,77,78,79,80] based on the most current scientific evidence. They do not specify, however, how conflicting situations should be addressed in practice. Older guidelines delegate more decisional power to pregnant patients’ autonomy due to scientific uncertainties, while more recent guidelines stress the importance of the evidence-based approaches leaning back towards medical paternalism [39].

Clinical practice guidance is mainly aimed at physicians, just briefly mentioning the involvement and role of nursing staff or other healthcare professionals, while oncology nurses in particular often encounter ethical issues due to complex patient care needs requiring expertise from multidisciplinary teams [48,81,82]. These nurses could play a significant role in directing patients to, or even delivering, initial counselling for patients affected by cancer while pregnant.

### 3.3. Balancing Maternalistic and Relational Approach to Evidence-Based, Personalised Patient Care

Caring for a pregnant cancer patient involves attempting to understand the reality in which this particular patient lives. This includes their way of seeing life, personal needs, expectations, commitments, desires and dreams for the future [83]. This approach to caring closely corresponds with key features found in models offered by care ethics (or ethics of care), relational ethics and nursing ethics. In these models, patient’s wellbeing is supported by providing patient-centred care, which is not limited to physical, medical and technical levels but has to include psychological, relational, social, moral and spiritual levels [84] and requires empathy from the healthcare team [64].

It is essential to note that patients place significant value on patient-centric care, which is perceived to be much broader than just treating a disease in a strictly technical sense [64,85]. It also includes taking care of a patient as a whole human being with compassion, empathy and recognition [45]. Despite the primary focus on a personalised care approach coming from the nurses, the care approach is also increasingly recognised among practicing physicians [64]. Furthermore, the medical maternalism approach to patient treatment and care provides the groundwork for clinical decisions taken or recommended for a patient based on a reasonable understanding of that person’s own preferences [50]. This is especially relevant in cases where a pregnant cancer patient lacks capacity, is seriously ill [33], is dying and/or being kept on life support for the benefit of the foetus [86] and is not able to participate in the medical decision-making process.

### 3.4. Considering Protection of the Vulnerable

This approach is often considered in light of responsibilities towards the unborn child [39,65] and underrepresented stakeholders, such as other children of a pregnant cancer patient and pregnant patients themselves. It has been reported that some physicians experience moral distress about perceived responsibilities towards the unborn child, who intuitively appears vulnerable and in need of protection [66]. However, a pregnant cancer patient and their developing foetus are interdependent and it would be inappropriate to view them as separate entities before the infant is born [33]. It is important to stress that for the expectant parents—pregnant cancer patient and their partner—the developing foetus is already a newborn, a “little person” that they are looking forward to being born. Therefore, the developing foetus might be regarded as vulnerable and in need of protection by its parents as much as healthcare professionals.

However, the potential vulnerability of an ill pregnant cancer patient should also be recognised. This does not mean that the pregnant cancer patient should be secured in a bubble or granted overprotection. However, it is important to recognise that the pregnant cancer patient, who is facing a potentially lethal disease, could be vulnerable to misinformation—alluding to “false hopes” [33]. This could lead to cancer treatment and pregnancy decisions that might not be treatment and care paths that the pregnant cancer patient genuinely wants to follow [87]. Some extensive studies have been carried out in exploring the vulnerabilities of pregnant women, such as when their developing foetus requires an intervention (e.g., foetal surgery) [68], suggesting that pregnant patient’s interest should not be overlooked [88].

### 3.5. Ensuring Reasonable and Just Resource Allocation

This is not a widely discussed topic in cancer treatment and care during pregnancy, but nevertheless it is important. Only one mention of this topic [75] was identified in clinical practice guidelines for cancer treatment during pregnancy review [39], but it has been raised as a concern in various circumstances, including facilitating “false hopes” among the patients and futile financial burdens to the patients and healthcare systems [33]. In some cases, a patient’s desire for motherhood can be so strong that it leads to unrealistic hopes of recovery and serves as a distraction from a potentially terminal illness, which could result in moral distress among the clinical team. While false hopes for recovery is a relevant concern for many seriously ill patients and not unique for cancer patients, it requires additional considerations when treating cancer during pregnancy.

It is, therefore, important to consider what responsibilities and obligations the healthcare systems and society at large have to pregnant cancer patients in terms of care, treatment and wider support. It is also essential to consider how resources for addressing the needs of pregnant cancer patients are obtained and distributed.

## 4. Ethics Checklist for Healthcare Professionals Attending to Pregnant Cancer Patients

Treating cancer during pregnancy can be emotionally intense and clinically challenging effort for healthcare professionals. They might have limited time and resources available for patient consultations, in-depth conversations and the identification of individual support needs. Therefore, an ethics checklist for clinicians is proposed below. It is informed by principles-based and relational care models, as well as five discussion themes identified earlier to assist the decision-making process when discussing treatment and care options with pregnant cancer patients (or their surrogate decision makers). This checklist was designed to assist healthcare professionals during clinical appointments, ensuring that potential and existing ethical issues in cancer during pregnancy treatment and care are addressed proactively, effectively and professionally.

### Ethics Checklist to Support Decision-Making Process in Treatment and Care of Pregnant Cancer Patients

- Perform an accurate clinical assessment to be able to discuss the disease prognosis, treatment intent (curative vs. palliative) and its impact on pregnancy.
- Identify the patient’s social and relational circumstances (e.g., spouse/partner/significant other; children; other relevant relationships; literacy and information comprehension level; occupation/employment situation; housing arrangements; socio-economic status; religion/spiritual/philosophical beliefs and needs; family/relational dynamics; gender identification; social roles important to the patient; etc.)
- Recognise the potential vulnerability of the pregnant cancer patient, take time to listen to patient’s concerns and fears, allow time to ask questions. Be informed about support services available for these patients.
- Recognise the developing foetus as a vulnerable entity and in need of protection by its parents as much as healthcare professionals. Take into consideration the gestational age of the foetus and the local legal requirements around pregnancy termination for medical reasons.
- Confirm and document patients’ decision-making capacity:

○ *If the patient is capable of consenting to medical treatment and interventions:*
  ○ Discuss with the patient their preferences regarding the medical decision-making process and communication with clinical team.
  ○ Discuss with the patient their preferences, expectations and perceptions about their desired cancer treatment and pregnancy outcome in their individual situation and care priorities, existing and desired advance care directives.
  ○ Support the patient with drafting, completing or updating the advance care directives, clearly document the patient’s intent for which circumstances they are applicable.
  ○ Identify other stakeholders involved in the clinical decision-making process (e.g., partner, parents, etc.) whose involvement is important to the patient.
  ○ Document all relevant information in the patient’s notes/electronic medical records.
○ *If the patient is not capable of consenting to medical treatment and interventions (unconscious, lacks capacity to make decisions):*
  ○ Identify the surrogate decision maker, ask about their own wellbeing and support needs, share information about available support.
  ○ Discuss the perceived patient and their caregiver(s)’ preferences with the surrogate decision maker, establish the expectations and perceptions about the desirable disease treatment and pregnancy outcome and care priorities, known/existing advance care directives, including the views and wishes the patient is known to have had expressed in the past.
  ○ Identify other stakeholders, who might need to be involved in the medical decision-making process (e.g., partner, parents, etc.) in order to establish the best interest of the patient and developing foetus.
  ○ If the patient is conscious, involve them in the conversation about their treatment and care options, where possible and practical.
  ○ Document all relevant information in the patient’s notes/electronic medical records.

- Upon confirming the decision-making capacity, share evidence-based information regarding treatment options and related clinical outcomes, expected short- and long-term toxicities for the patient and the developing foetus.
- Inform the patient/surrogate decision maker about ongoing cancer during pregnancy research and available options for participation in clinical trials.
- Involve a multidisciplinary team that includes different medical specialties with expertise in care of pregnant patients with cancer and other healthcare professionals, such as nurses, psychologists, social workers, hospital ethics committee/ethics advisory board, ethical and spiritual care providers, etc.
- Obtain a written consent to treatment and/or care plan (if required by local regulations), allowing adequate time for decision making, following the discussion on available cancer and pregnancy management options.
- Where written consent to treatment/care plan is not a routine or mandatory requirement, allow adequate time for decision making, following the discussion on available cancer and pregnancy management options and document it in the patient’s notes/electronic medical records.
- Clearly document patient’s/surrogate decision maker’s decisions, concerns and explanation given on treatment and care in patient’s notes/electronic medical records.
- Periodically review changes in patient’s treatment and care plan, updating consent documentation as per local legal requirements and professional guidance.
- Seek consultation with other hospital/care facility teams (hospital ethics committee/ethics advisory board, social services, patient financial support, patient counselling, etc.) if available healthcare resources are not adequate for handling a particular patient’s case, if patient’s or surrogate decision maker’s treatment/care preferences are futile and might result in significant financial burden to the healthcare system or themselves.
- Ensure that the patient/surrogate decision maker are aware of an option to request a second opinion from another doctor/multidisciplinary team without retaliation from the treating doctor/team or administration.
- Should patient/surrogate decision makers refuse treatment or suggested care pathway, seek to understand the reasons behind it, be ready to answer questions and give time to consider the options without retaliation from the treating doctor/team or administration.
- Identify existing and potential concerns within the clinical team based on legal considerations, political leaning, religious beliefs and personal preferences; seek reconciliation of such concerns in a structured manner (e.g., moral case deliberation, clinical ethics consultation, ethical counselling, etc.)
- Acknowledge the rights and their legal/professional limits for clinical and supporting team members to exercise conscientious objections (e.g., administering treatment to a pregnant patient that can potentially harm the foetus, carrying out abortion/pregnancy termination procedures, proving post-abortion care to cancer patients, etc.), constructively engage concerned team members, seek council with the legal team, ethics consultation service, senior management, etc., to ensure that patient safety, continuity of treatment and care are not compromised due to moral objections leading to staff shortage.

## 5. Conclusions

This concept paper proposes a theoretically grounded framework to support ethical and patient-centred care, treatment and counselling of pregnant cancer patients. The five themes summarising the key ethical considerations that should be taken into account by healthcare providers while discussing treatment and care options with these patients are reflected in the proposed ethics checklist for supporting the decision-making process when attending to pregnant cancer patients. Both the framework and the ethics checklist were developed to assist the decision-making process by facilitating the early identification of possible ethical concerns that may arise in cancer during pregnancy care.

With this work, the authors expect to launch the debate on this under-looked but very important domain of pregnant cancer patients care. Nevertheless, further work is needed to build comprehensive, structured and patient-centric ethical guidance for cancer treatment during pregnancy. The emerging evidence that some cancer treatments can be given to pregnant patients without significant adverse effects on a developing foetus and a future child tends to overshadow the importance of holistic approaches to patient care [39]. Meanwhile, the voices of affected patients and their partners/family members are still missing from the mainstream debate as there are only few studies exploring the experiences and concerns of cancer patients who are also parents [89,90,91,92] and pregnant patients who experience critical health conditions [2,93,94,95]. Furthermore, there are just a few studies exploring the practices and clinicians’ attitudes towards cancer treatment in the course of pregnancy [96,97,98] and even fewer studies exploring patient experiences and attitudes when diagnosed with cancer while pregnant [99]. A more in depth understanding of experiences, attitudes and approaches held by different stakeholders to cancer care during pregnancy would help to form a comprehensive ethics guidance for healthcare professionals, including nurses and patient support service providers. Therefore, future work would require interdisciplinary collaboration between physicians, nurses and other healthcare professionals, as well as social scientists, psychologists, ethicists, spiritual care providers and patient representatives. Having a well-grounded clinical practice guidance and practical tools for ethical, patient-centred care of pregnant cancer patients would provide educational and practical background for healthcare professionals for addressing ethical issues arising in their practice proactively, effectively and professionally.

## Figures and Tables

**Table 1:** Considerations for treating cancer during pregnancy.

Type of Malignancy	Modes of Treatment	Considerations for Pregnant Patients	Considerations for the Foetus
Breast cancer [19,20]	Surgery (safe throughout pregnancy), radiotherapy (contraindicated in pregnancy), chemotherapy (second and third trimester), hormonal/endocrine therapy (contraindicated), immunotherapy (contraindicated, PD-1/PD-L1 pathway could result in immune response against the foetus), targeted therapy (contraindicated with exception of trastuzumab, which may be used in the first trimester under close monitoring).	Physiological breast changes should be considered, delaying reconstruction surgery after delivery. Higher risk of pregnancy complication cannot be excluded.	Increased risks of stillbirths, small gestational weight, preterm delivery, neonatal mortality. No significant impairment after exposure to chemotherapy. Prematurity correlated with worse cognitive outcome irrespective of cancer treatment.
Thyroid cancer [21,22]	Surgery (second trimester or after delivery), endocrine therapy (LT4 therapy should start immediately after surgery), radioactive iodine (contraindicated in pregnancy and breastfeeding), immunotherapy with tyrosine kinase inhibitors (TKIs) is not well studied.	Calcium and vitamin D supplementation, hypothyroidism should be avoided by correct supplementation of thyroxine. No evidence to support pregnancy termination.	Thyroid hormone deficiency can cause severe neurological disorders.
Cervical cancer [7,23,24]	Hysterectomy (in advanced cases, can be combined with a caesarean delivery or performed post-partum, otherwise not compatible with pregnancy), cold knife conization (risk of premature birth), radical trachelectomy/cervicectomy (risk of premature birth), chemotherapy (second and third trimester), radiotherapy (contraindicated).	Caesarean section is preferred delivery method, especially in advanced cases. Fertility preservation in advanced cases might not be possible. Chemotherapy is not recommended beyond 35 weeks of gestation to allow maternal and foetal bone marrow recovery before delivery.	Chemotherapy can affect foetal eyes, genitals, hematopoietic system, nervous system, foetal growth. Single cases of bilateral hearing loss and rhabdomyosarcoma have been reported.
Other gynaecological cancers (vulvar, vaginal, endometrial, ovarian cancer, ovarian masses with low malignant potential) [7]	Laparoscopic surgery (feasible throughout pregnancy, not longer than 90–120 min), surgery (decided upon individual cases), chemotherapy (second and third trimester), radiotherapy (contraindicated), systemic therapies not well studied.	Caesarean section is a preferred delivery method, especially in advanced cases. In cases of advanced epithelial ovarian cancer, pregnancy termination should be considered in the first half of pregnancy. Chemotherapy is not recommended beyond 35 weeks of gestation to allow maternal and foetal bone marrow recovery before delivery.	If possible, delivery should not be induced before 37 weeks to allow foetal maturity. Breastfeeding should be avoided with ongoing chemotherapeutic, endocrine and targeted treatment.
Lymphomas (Hodgkin lymphoma and non-Hodgkin lymphoma) [25,26]	Chemotherapy (second and third trimester), radiotherapy (conflicting data), immunotherapy (limited data)	Deferring therapy until after delivery does not always affect maternal outcomes and can be considered. Pregnancy termination can be considered in the first trimester. Patients receiving antenatal therapy have more obstetric complications (preterm contractions and preterm rupture of membranes).	No gross foetal malformations or anomalies have been reported. Low gestational age and admissions to NICU did not differ between neonates exposed and not exposed to chemotherapy. Those exposed to chemotherapy had lower birth weight.
Melanoma [27,28]	Excisions (throughout pregnancy—safe and necessary), targeted therapies (BRAF inhibitors) and checkpoint inhibitors (anti-PD1 and anti-CTLA4) may be teratogenic.	Relationship between pregnancy and melanoma should not be ruled out. Some reports suggest poorer prognosis for pregnant patients, but evidence is inconclusive.	No evidence that melanoma diagnosis will have adverse effected on the foetus. Melanoma accounts for 30% of metastatic spread to the placenta. This does not mean that the foetus will be affected.
Brain tumours [29,30]	Surgery, chemotherapy, radiotherapy—only limited data available due to rarity of the condition.	Delivery recommended after 34 weeks of gestation to allow foetal maturity. Caesarean delivery recommended.	No known foetal complications. Steroids for foetal lung maturation might be needed if early delivery is needed due to deteriorating maternal condition.
Lung cancer [31,32]	Chemotherapy (second and third trimester), targeted therapies—only limited data available due to rarity of the condition	Increased risk of lung infections. Case reports suggest that lung cancer is diagnosed at advanced stages in pregnancy and prognosis is poor.	No adverse outcomes data reported. Due to advanced stage of maternal cancer, there might be a metastatic spread to the placenta. This does not mean that the foetus will be affected.

**Table 3:** Foundational discussion themes for ethical, patient-centred care framework for cancer treatment during pregnancy.

Discussion Themes: Ethical, Patient-Centred Care of Pregnant Cancer Patients
Recognising relational context of individual patient’s autonomy and supporting it through caring, patient-focused approach to build trusted relationships between the pregnant cancer patient and the healthcare team
Balancing maternal and foetal beneficence can be supported by caring, patient-focused and maternalistic approach to patient care with best interest of the patient and their foetus in mind
Balancing maternalistic and relational approach with evidence-based, personalised patient care while attempting to understand individual realities of pregnant cancer patient
Considering protection of the vulnerable in a light of responsibilities towards the unborn child and underrepresented stakeholders, such as other children of a pregnant cancer patient and pregnant patients themselves
Ensuring reasonable and just resource allocation to avoid giving pregnant cancer patient false hopes and creating futile financial burdens

## Data Availability

All available data are reported in the article. There are no additional data available.
